# Detection of Parasite-Specific DNA in Urine Sediment Obtained by Filtration Differentiates between Single and Mixed Infections of *Schistosoma mansoni* and *S. haematobium* from Endemic Areas in Ghana

**DOI:** 10.1371/journal.pone.0091144

**Published:** 2014-03-14

**Authors:** Nilanjan Lodh, Jean M. Naples, Kwabena M. Bosompem, Joseph Quartey, Clive J. Shiff

**Affiliations:** 1 Department of Molecular Microbiology and Immunology, Johns Hopkins Bloomberg School of Public Health, Baltimore, Maryland, United States of America; 2 Parasitology Department, Noguchi Memorial Institute for Medical Research (NMIMR), Legon, Accra, Ghana; Food and Drug Administration, United States of America

## Abstract

Differential diagnosis of *Schistosoma mansoni* and *S. haematobium*, which often occur sympatrically in Africa, requires both urine and stool and the procedures are low in sensitivity. The standard diagnostic tests, such as Kato-Katz (KK) for *S. mansoni* eggs and presence of haematuria for *S. haematobium* both lack sensitivity, produce false-negative results and show reduced accuracy with decreasing intensity of infection. The need for a single diagnostic test with high sensitivity and specificity for both parasites is important as many African countries are implementing Mass Drug Administration (MDA) following recommendations of the World Health Organization (WHO). Eighty-six samples of urine sediment obtained by filtration were collected from a group of 5–23 years old people from an endemic area of southern Ghana. DNA was extracted from the urine sediment on filter paper from which a species-specific repeat fragment was amplified by polymerase chain reaction (PCR) with specific primers for *S. mansoni* and for *S. haematobium*. Additionally, all participants were tested by KK (stool) and dipstick for haematuria. Diagnostic parameters for all three tests were analyzed statistically. Amplification of species-specific DNA by PCR showed much higher sensitivity (99%–100%) and specificity (100%) compared to KK and haematuria (sensitivity: 76% and 30% respectively) for both schistosome species. The same pattern was observed when the data were stratified for age group and sex specific analysis. In addition PCR amplification detected DNA from 11 individuals infected with both parasites who were negative by KK and haematuria. This approach of detecting parasite specific DNA from either or both species in a single urine specimen is a practical advantage that avoids the need for two specimens and is more effective than standard tests including those based on serology. This promises to improve the effectiveness of surveillance of MDA control programs of schistosomiasis.

## Introduction

The true disease prevalence of schistosomiasis in endemic areas of sub-Saharan Africa remains unclear due to poor sensitivity and specificity of current standard parasitological tests for diagnosis. In this region, schistosomiasis is caused by two major human species *Schistosoma mansoni* and *S. haematobium*, often concurrent in the human population [1]. This polyparasitism is common in schistosome endemic areas [2], which raises the problem of an accurate and specific diagnosis most importantly when infections are asymptomatic. Here we show that detection of species-specific DNA in urine with single or mixed species infections can differentiate between these two sympatric parasite species in people from Ghana.

Previous studies showed that parasite specific DNA can be detected in urine of infected persons in the case of *Plasmodium* [3] and schistosomes [4], [5]. For schistosomes the source of DNA is the adult worm, which sheds tegument at regular intervals and detection of such parasite specific DNA from urine proves the presence of parasite [6]. Demonstration of schistosome parasite specific DNA in urine has been shown by multiple authors in recent years. For *S. haematobium*, Ibironke et al. [6] in a detailed study on latent class modeling demonstrated that detection of a species-specific DNA fragment from urine sediment obtained by filtration is the best estimator of true infection. They also concluded that this approach is closer to the actual “gold standard” for schistosome diagnosis than presence of eggs, antigen capture or serology. Furthermore with *S. haematobium*, DNA becomes undetected two weeks after drug treatment in Nigerian people [4]. Even for *S. mansoni*, species-specific DNA detection from urine sediment obtained by filtration from Zambia proved to be more sensitive and specific than KK. A serological approach, such as the Circulating Cathodic Antigen test (CCA), which has reasonable sensitivity for *S. mansoni* is inadequate to detect evidence for *S. haematobium* [5]. Having demonstrated these advantages, it is appropriate now to consider mixed schistosome infections.

Differentiating between species can be tedious, as it involves examining both urine and stool. Logistically and technically such tasks are time consuming and these procedures are often inadequately sensitive [4], [5]. More specifically for *Schistosoma haematobium* egg passage in the urine is affected by circadian rhythms as well as bladder wall hyperplasia making egg detection in urine so inadequate that it is difficult to assess the prevalence of infection among adult population [4], [7]. The situation is worse with *S. mansoni* as standard test, the Kato-Katz (KK), has low sensitivity and gives a poor variable estimate of the worm burden [8], [9]. In fact, detection of eggs in urine and/or stool is not a gold standard, because as has been clearly demonstrated absence of eggs is not necessarily indicative of absence of infection [4], [5]. Detection of parasite specific DNA in urine or bodily fluids indicates the presence of the actual parasite even when eggs or other products such as antigens are not always detectable [3], [7]. Among other effects, schistosomiasis is also an inflammatory disease, as *S. mansoni* causes intestinal inflammation with advanced hepatic fibrosis [10], whereas *S. haematobium* is associated with bladder and kidney inflammation, bladder cancer, and infertility [11]–[14]. Because of the different organ system involved and the pathological implications of single or mixed infections, it is important to differentiate accurately between them.

In this study, we adapted the use of an extensive repeat sequence of the schistosome genome, which comprises ∼12% of the genome [15], [16]. The sequences are non-coding, short tandem repeats (∼121 bp) and specific for *S. mansoni* and also for *S. haematobium*. Hamburger and others have successfully demonstrated the amplification of such repeat fragments from snails [15], [16]. More recently, parasite species-specific repeat DNA detection has been done for *S. mansoni* from human serum and feces [17] and also from whole urine [18] and more recently from urine sediment filtered through coarse filter paper [5]. *S. haematobium* repeat fragments also have been successfully amplified from human urine sediment [4].

The approach for this study was multifold. Samples were collected from an endemic area of Ghana, where both parasite species live sympatrically. All samples were tested for eggs by Kato-Katz (KK) and for haematuria (blood in urine) as determinants of *S. mansoni* and *S. haematobium* infection. Haematuria was merely a sign that *S. haematobium* might be present, and had been shown to be reasonably associated with the infection [3]. The possibility of identification of DNA of both parasite species by PCR from the same urine sample will simplify the collection of samples considerably. The efficacy and accuracy of PCR against standard KK and haematuria, both separately and combined, were evaluated statistically. Also nine chosen mixed infection samples were sequenced to rule out any cross amplification by the species specific primers.

## Materials and Methods

### Study location and population

The study was conducted at Tomefa, a community in the Weija Lake area (the outflow lake of the Densu River) of Ga South District of Greater Accra Region in Ghana. This location was chosen because no testing or mass drug treatment for human schistosome infection was done there in the last 12 months. Moreover this water body is the major source of water for all household activities and also for occupational purposes for local residents. For these reasons there was a higher chance of local individuals to be exposed to schistosome parasites as the water harbors *Bulinus globosus* and *Biomphalaria* sp. intermediate host snails. We detected high proportion of mixed schistosome species infection from this area.

This study involved 86 individuals aged from 5–23 years. This age group (children and young adults) was chosen because of the high prevalence and intensity of schistosome infection in young compared to older individuals. Specimens were only taken from adults aged 18 years or more who consented to participate and from children following parental consent.

### Study Design and Ethical Clearance

Approval for the diagnostic research was provided by the Noguchi Memorial Institute for Medical Research (NMIMR) IRB 043/12-13 (Approval S1). De-identified specimens were collected and brought to Baltimore, Maryland, where all samples carried only numbers that were associated with specimen age, sex and diagnostic information. Participants were fully briefed on the importance of schistosomiasis and written informed consent was collected from adults and from parent or guardian in case of child. Participants found infected parasitologically were offered treatment with praziquantel through the NMIMR and the local health centre. Participation was entirely voluntary.

Participants were provided with plastic cups for urine collection taken between 10:00 am and 2:00 pm for maximum passage of eggs [7] and approximately 100 ml urine was collected. Sealable cups were provided for stool, which was collected the following day. Urine samples were evaluated for colour, pH, and specific gravity and for presence of protein, glucose bilirubin and haematuria with Hemastix (Bayer, Elkhart, IN). Stool was examined parasitologically using the KK method and reported positive or negative according to the presence or absence of *S. mansoni* eggs.

### Urine preparation for DNA detection

Approximately 50 ml urine was filtered through a cone shaped 12.5 cm Whatman No. 3 filter paper disc (Whatman International, Maidstone, England). Each filter paper was marked with unique identification number of the participant. The cone was set in a plastic funnel to pass the urine. To avoid contamination, plastic cups and funnels were used only once. After filtration, the paper disc was opened and air dried under a fly-proof net and then individually packed with desiccant in a plastic sheath and transported to Baltimore for DNA extraction and species-specific DNA amplification by PCR.

### DNA extraction from urine

Urine sediment obtained by filtration captured single type or mixed schistosome DNA depending on the infection status. The inner quadrant of the folded marked cones was used to extract the DNA. The quadrant was cut and 12–15 punches were made (∼1 mm in diameter) by regular paper punch before being subjected for DNA extraction. The paper punch and scissor was cleaned with 10% bleach solution and dried before processing any new sample to avoid contamination. For each specimen, 12–15 discs of 1 mm size were placed into a 1.5 mL eppendorf tube containing 600 μL nuclease-free water. The tubes were incubated at 95°C for 10 min and then put on a rotator at room temperature for overnight (16–18 hrs). The water solution was then transferred to a Qiagen QIAmp 2 mL column tube. DNA was precipitated and concentrated using the QIAmpDNA Blood Mini Kit (Qiagen, MD) according to the manufacturer's protocol. DNA concentration was measured using the NanoDrop ND-1000 spectrophotometer (NanoDrop Technologies, USA) and stored at −20°C for future use.

### PCR test for *S. mansoni* and *S. haematobium*

Polymerase chain reaction (PCR) was administered on all 86 samples to detect single or mixed schistosome infection. For *S. mansoni* PCR was carried out by using forward primer SmPF (5' GATCTGAATCCGACCAACCG 3') and reverse primer SmPR (5' ATATTAACGCCCACGCTCTC 3'), which were previously described by Pontes [17]. It amplified 110 bp fragment from a highly repeated 121 bp region of *S. mansoni* described by Hamburger [16]. The repetitive fragment constitutes at least 12% (600,000 copies per cell) of the *S. mansoni* genome. For *S. haematobium* two species specific primers ShDra1F (forward:5′- GATCTCACCTATCAGACGAAAC-3′) and ShDra1R (reverse: 5′-TCACAACGATACGACCAAC-3′) were used, which were previously designed by Hamburger and others [15] for specific amplification of 121 bp *Dra* 1 repeats of *S. haematobium*. Both sets of primers were designed based on repeats occur in different region of genomes of these two schistosome parasites.

Amplification reactions were carried out in 10 μL volume with a positive and negative control. For *S. mansoni* stool samples positive for egg by KK were used as positive control, whereas for *S. haematobium* samples positive for haematuria were used as positive control. Water was used as negative control throughout all PCR run. 2 μL of DNA (concentration: 4–6 ng/μL) served as PCR template in a reaction volume that comprised 5 μL of 2X PCR Master Mix (Promega, Madison, WI), 0.5 μL (10 μM) of each of the amplification primers, 1.5–2 μL of 25 mM MgCl_2_ and sterilized water to complete the final volume. The amplification procedure for *S. mansoni* was initial denaturation at 95°C for 10 min, followed by 35 cycles of 95°C for 30 sec, annealing temperature of 60°C for 90 sec, and elongation at 72°C for 30 sec, followed by a final extension step at 72°C for 5 min. For *S. haematobium* there an activation/denaturing step of 10 min at 95°C, followed by 33 cycles of 95°C for 30 sec, annealing temperature of 53°C for 90 sec, and elongation at 72°C for 1 min, followed by a final extension step at 60°C for 5 min. Electrophoresis of 4 μL amplified PCR product was carried out in a 2% agarose gel stained with ethidium bromide (10 mg/μL) with the help of 50 bp ladder (New England BioLabs Inc., Ipswich, MA) to estimate band sizes.

### Sequencing of mixed infection samples

Nine mixed infection samples were selected for sequencing in order to demonstrate that differentiation between the species in a single specimen was possible. The specimens were positive for *S. mansoni* by KK and for haematuria. To prepare for sequencing all nine samples were sequenced individually by the two sets of primers specific for *S. mansoni* and *S. haematobium*. PCR products were cleaned with ExoSAP-IT (Affymetrix Inc., Cleveland, OH) and were provided for sequencing. Sequencing was done by the genetic resources core DNA analysis facility (GRCF, The Johns Hopkins University). Subsequently all forward and reverse sequences were searched for appropriate schistosome species match through NCBI BLAST.

### Statistical analyses

For statistical analysis data were converted to numerical values (1 =  positive and 0 =  negative) for KK, haematuria and PCR result of both *S. mansoni* and *S. haematobium*. In columns of all tables data were represented as KK_*S. mansoni*, PCR_*S. mansoni*, haematuria_*S. haematobium*, PCR_*S. haematobium* and Mixed infection detected by PCR. The positive and negative case assessment was based on following predictions. 
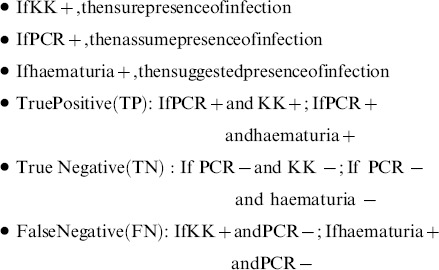



The sensitivity, specificity, positive predictive value (PPV), negative predictive value (NPV) was calculated using MedCalc 12.4.0 (MedCalc Software, Belgium). Disease prevalence was based on number of positive cases by each diagnostic test out of total samples diagnosed. Kappa value and Bowker's Symmetry [20] were calculated (JMP v9, SAS Institute Inc., Cary, North Carolina) to determine the agreement between two diagnostic tests. For the current study it was calculated for KK vs. PCR for *S. mansoni* and haematuria vs. PCR for *S. haematobium*. Participants were divided into two age groups (A = 5–12 yrs and B = 13–22 yrs) and were evaluated for positivity and negativity of each diagnostic test out of total number of samples had been analyzed. In addition sex specific evaluation (JMP v9, SAS Institute Inc., Cary, North Carolina) was also done to establish the robustness of parasite specific DNA detection and its importance to detect mixed infection in an endemic area.

## Results

The objective of this research was to investigate the efficacy of detecting parasite specific DNA amplified from urine sediment obtained by filtration from people infected with both *S. mansoni* and *S. haematobium* either singly or together. Specimens were examined in the field and in the lab, with stool examination (KK smear), urine examination for haematuria and parasite specific DNA detection by PCR. The results are presented accordingly.

### Detection of *S. mansoni* infection

All 86 stool samples were evaluated for *S. mansoni* by KK of which 57 were true positives (TP), 11 were true negatives (TN) and importantly 18 were false negatives (FN; Table 1). In comparison, PCR for *S. mansoni* detected 74 people as TP and only 1 as FN (Table 1). The trend was evident for disease prevalence, which was 66% for KK and 86% for PCR (Table 2). Diagnostic parameters for sensitivity and specificity were calculated for KK, 76% sensitivity (95% CI: 65%–85%), whereas for DNA amplification 99% sensitivity (95% CI: 93%–99%) (Table 2). The specificity and positive predictive value (PPV) remained 100% for both methods (Table 2). The negative predictive value (NPV) for stool smear was 38% due to high false negatives for KK (Table 2). To validate the existence of species-specific DNA, four specimens KK positive were sequenced and authenticated by NCBI BLAST, which matched perfectly with *S. mansoni* genome. The agreement statistics including Kappa coefficient and Bowker's symmetry were calculated by comparing KK and PCR test for *S. mansoni*. Kappa determined the agreement between two tests based on the following parameters (−1 =  negative association, 0 =  random, +1 =  complete agreement). The statistical symmetry of disagreement between these two testes was calculated by Bowker's symmetry. This test checked for symmetry in 2-way tables and the test decision was based on *X^2^*approximation of the distribution of the test statistic [19]. A significantly low level of agreement was detected between KK and PCR (kappa: 0.43; 95% CI: 0.23–0.62; P<0.05; Table 3). The Bowker Symmetry was significantly different from random (P<0.05) for the comparison, indicating that test positives by KK were highly unlikely to be the same with PCR (Table 3).

**Table 1:** Frequency of positive and negative infection for *Schistosoma mansoni* and *S. haematobium* infected people from Ghana evaluated by Kato-Katz (KK), haematuria and polymerase chain reaction (PCR).

Diagnostic Test	True Positive (TP)	False positive (FP)	True Negative (TN)	False Negative (FN)	Total
KK*_*S. mansoni*	57 (66%)	0	11 (13%)	18 (21%)	86
PCR_*S. mansoni*	74 (86%)	0	11 (13%)	1 (1%)***	86
haematuria**_*S. haematobium*	21 (24%)	0	16 (19%)	49 (57%)	86
PCR_*S. haematobium*	70 (81%)	0	16 (19%)	0	86
PCR_*S. mansoni* and *S. haematobium* combined	82 (95%)	0	3 (4%)	1 (1%)***	86

Combined PCR results mentioned at the bottom.

* KK =  Stool examination.

** Haematuria  =  Blood in urine.

*** The single false negative was repeated with several extractions. There was no amplification detected.

**Table 2:** Estimation of disease prevalence, sensitivity, specificity, predictive values and likelihood ratios byPCR against standard parasitological tests* for current study for identifying mixed schistosome infection from Ghana.

Diagnostic Test	Disease Prevalence**	Sensitivity (95% CI)	Specificity (95% CI)	Positive Predictive Value (PPV)	Negative Predictive Value (NPV)
KK_*S. mansoni*	66%	76% (65%–85%)	100% (71%–100%)	100%	38%
PCR_*S. mansoni*	86%	99% (93%–99%)	100% (71%–100%)	100%	92%
haematuria_*S. haematobium*	24%	30% (20%–42%)	100% (79%–100%)	100%	25%
PCR_*S. haematobium*	81%	100% (95%–100%)	100% (79%–100%)	100%	100%

*Standard parasitological tests  = KK for *S. mansoni* and haematuria for *S. haematobium*.

**Disease prevalence  =  Number of positives by each test out of total number of samples had been analyzed.

**Table 3:** Agreement statistics were calculated for KK, haematuria and PCR.

Diagnostic Test	Kappa Coefficient			Bowker's Symmetry test*	
	Kappa value	95% CI	P valueψ	Symmetry of Disagreement	P valueψ
KK vs. PCR_*S. mansoni*	0.43	0.23–0.62	0.0001*	15.21	0.0001*
Haematuria vs. PCR_*S. haematobium*	0.14	0.06–0.22	0.0059*	49	0.0001*

*Bowker's Symmetry test  =  this test checks for symmetry in 2-way tables and the test decision is based on a *X* ^2^ approximation of the distribution of the test statistic.

ψ = α level was set at 0.05.

* = Significant.

Specimens were stratified into two age groups and by sex and results were analyzed similarly to test the reproducibility of the results. The results showed compatibility with the overall analysis.

### Detection of *S. haematobium* infection

In the field, only dipstick (haematuria) was used to indicate *S. haematobium* infection and for determination of TP for haematuria (21), which was much lower in comparison to *S. haematobium* specific DNA detection by PCR (70; Table 1). Moreover haematuria yielded far more FN cases (49), whereas none by PCR (Table 1). This is to be expected as haematuria is a non-specific indicator and only used as surrogate for schistosomiasis haematobia (Table 2). The sensitivity of PCR technique (100%) was significantly higher than haematuria (30%) and yielded higher infection prevalence (81%; Table 2) among studied individuals. Moreover PCR showed 100% PPV and NPV (Table 2). To validate the existence of species-specific DNA, four specimens KK positive were sequenced and authenticated by NCBI BLAST which matched perfectly with *S. haematobium* genome. The primers did not amplify any sequence other than *S. haematobium*.

The kappa agreement statistics indicated that PCR performed significantly better in detecting *S. haematobium* infection than haematuria (kappa: 0.14; 95% CI: 0.06–0.22; P<0.05; Table 3). The symmetry of disagreement was also significantly higher (49; P<0.0.5), which indicated that PCR scored more positives, especially when infection are asymptomatic and cannot be determined by haematuria (Table 3). Analyses related to two age groups and gender also showed similar outcomes as diagnostic parameter analyses. Parasite DNA detection identified more positive infection in both age groups (Group A = 48 and Group B = 22) in comparison with haematuria, which identified only 11 and 10 (Table 4). In addition high number of female and male positive cases were also detected by PCR (33 and 41), whereas haematuria detected only 7 and 14 (Table 4).

**Table 4:** Effectiveness of KK, haematuria and PCR as diagnostic tests to distinguish single and mixed schistosome infection from Ghana across different age groups and for both genders.

Diagnostic Test	Age Group A (5–12yrs)		Age Group B (13–22yrs)		Female		Male	
	Positive*	Negative**	Positive	Negative	Positive	Negative	Positive	Negative
KK_*S. mansoni*	35 (41%)	21	22 (26%)	8	25 (29%)	14	32 (37%)	15
PCR_*S. mansoni*	48 (56%)	8	26 (30%)	4	31 (36%)	8	39 (45%)	8
Haematuria_*S. haematobium*	11 (13%)	45	10 (12%)	20	7 (8%)	32	14 (16%)	33
PCR_*S. haematobium*	48 (56%)	8	22 (26%)	8	33 (38%)	6	41 (48%)	6
PCR_*S. mansoni* and *S. haematobium* combined	54 (63%)	2	28 (33%)	2	37 (43%)	2	45 (52%)	2

This table tests the reproducibility of result between tests by segregating into groups based on age or sex.

*Positive  =  Number of positives by each test out of total number of samples had been analyzed.

**Negative  =  Number of negatives by each test out of total number of samples had been analyzed.

### Detection of mixed schistosome infection

This study found people infected with both schistosome species detected from extracted DNA from a single urine specimen. Parasite specific DNA detection found 82 people were infected either by single species or by mixed species. Only one sample was FN by PCR in case of *S. mansoni* (no DNA was detected from one specimen with a *S. mansoni* egg, Table 1). In detail DNA detection found that 12 individuals were infected only by *S. mansoni*, whereas eight only by *S. haematobium* (Table 5). A total of 62 out of 86 people were detected with mixed infection. Importantly 11 people who had no sign of infection parasitologically were detected positive (DNA amplification) for both schistosome species (Table 5). Age stratified analysis also showed more positive infection detection (54 and 28) for both age groups in case of combined PCR analysis for both species (Table 4). In addition, parasite specific DNA detection test was more sensitive than KK and haematuria for both female and male both positive and negative (Table 4).

**Table 5:** Detailed numerical distribution of single and mixed schistosome parasite infected people from Ghana diagnosed by KK, haematuria and PCR*.

Diagnostic tests	Infection type			
	*S. mansoni* only	*S. haematobium* only	Mixed infection	No sign of infection
KK_*S. mansoni*	9	-	47 out of 62	0
PCR_*S. mansoni*	12	-	-	-
haematuria_*S. haematobium*	-	1	20 out of 62	0
PCR_*S. haematobium*	-	8	-	-
Mixed infection detected by PCR	-	-	**62**	**11**

* Important findings by PCR are highlighted. Areas not applicable remain blank.

## Discussion

There is strong evidence that detection of parasite specific DNA in urine is more sensitive and specific than processing urine or stool by standard methods [4]–[6]. The current work has now demonstrated that it is feasible to detect single or mixed infections of both *Schistosoma haematobium* and *S. mansoni* from a single urine specimen and thus distinguish either a uniparasitic or polyparasitic infection with the same high quality. The savings in time and cost are high, because stool collections are cumbersome, require two visits, to provide receptacles and then pick them up. Stool also requires elaborate preparations, protective clothing, air extraction equipment to mange odour and the hazard of dealing with faeces. Both the Kato-Katz and urine filtration tests are low in sensitivity and as demonstrated here, miss a high proportion of cases. The multi species detection approach is also robust in that it avoids cross amplification by primers and identifies the specific parasite responsible for the disease. This technique is important for long term success of parasite control by Mass Drug Administration (MDA) intervention because of the need to detect low intensity infections that persist even after treatment has occurred [20]. People with low parasitaemia still pass viable schistosome eggs and the emerging miracidia are efficient in locating the intermediate host snails [21] and maintaining the cycle. This increases the possibility of quick re-establishment of disease transmission. The methods described here will simplify the process of multi species diagnosis in an effective way to improve the surveillance and monitoring system.

The need for a specific and accurate diagnostic test for schistosomiasis is important as overall, the current incidence of schistosomiasis does not show any signs of decline [22]. In addition the present disability-adjusted life year (DALY) system of the World Bank and the World Health Organization (WHO; [23]) underestimates morbidities, including polyparasitism in *Schistosoma* endemic areas [24]. This problem is illustrated by the fact that of those that are carrying both parasites (62), 11 tested negative by both KK and haematuria (Table 5). These 11 people are positive by species-specific DNA detection, which proves that asymptomatic infections are important. Without a sensitive and effective diagnostic test for the asymptomatic infections the accurate estimation of schistosomiasis disease prevalence is not possible [25].

Without highly sensitive, specific tests, it will be difficult to ensure the objective control of schistosomiasis. This study addresses the importance of sensitivity of detecting species-specific DNA by PCR. Optimization of the above stated approach for use in the field is a future goal. Importantly there is added cost involved in employing PCR based DNA detection in the field, but in most endemic countries and in developing countries the facilities for PCR do exist. The facilities can also be progressively introduced in the endemic areas as the merit of this diagnosis test has been demonstrated by the current study. The species-specific DNA detection by PCR appears to be more sensitive than KK thick smears and haematuria for diagnosis of *S. mansoni* and *S. haematobium* from a single source of DNA, which is efficiently collected and filtered through a single filter paper. It is therefore can be an important tool for rapid identification of multi schistosome species infected individuals before and after the chemotherapy.

## Supporting Information

Approval S1 (PDF)
