# Supplementary material for: Detection of Parasite-Specific DNA in Urine Sediment Obtained by Filtration Differentiates between Single and Mixed Infections of Schistosoma mansoni and S. haematobium from Endemic Areas in Ghana
Source: PLoS One. 2014 Mar 14;9(3):e91144. doi: 10.1371/journal.pone.0091144 (PMC3954594; doi:10.1371/journal.pone.0091144)
Supplement: Approval S1 — (PDF) [file pone.0091144.s001.pdf]

NOGUCHI MEMORIAL INSTITUTE FOR MEDICAL RESEARCH

Established 1979 A Constituent of the College of Health Sciences
University of Ghana

Phone: +233-302-916438 (Direct)
+233-289-522574
Fax: +233-21-502182/513202
E-mail: nirb@noguchi.mimcom.org

NMIMR-IRB
P. O. Box LG 581
Legon, Accra
Ghana

My Reference: DF 22

November 15, 2012
Professor K. M. Bosompem
Noguchi Memorial Institute for Medical Research, University of Ghana
P. O. Box LG 581
Legon, Accra

RE: Our Study # 043/12-13 **At:** NOGUCHI MEMORIAL INSTITUTE FOR MEDICAL RESEARCH-IRB

Dear Professor K. M. Bosompem:

Meeting Date: 11/14/2012 **At:** NOGUCHI MEMORIAL INSTITUTE FOR MEDICAL RESEARCH-IRB
Protocol Title:

Field Testing for the detection of schistome specific DNA using LAMP amplification

This is to advise you that the above referenced Study has been presented to the Institutional Review Board, and the following action taken subject to the conditions and explanation provided below.

Internal #: New Appl
Expiration Date: 11/13/2013
On Agenda For: Initial Submission
Reason 1: **Reason 2:**
Description: Date Received- 10/23/2012
IRB ACTION: Approved
Condition 1:
Action Explanation:

Yours Sincerely,

Helena Baidoo
IRB Administrator
NMIMR-IRB
cc: Dr. Joseph Hamburger , Hebrew University of Jerusalem (HU), Prof. Clive Shiff, PhD, John Hopkins Bloomberg School of Public Health
